# Supplementary material for: Proximity-driven site-specific cyclization of phage-displayed peptides
Source: Nat Commun. 2024 Aug 24;15:7308. doi: 10.1038/s41467-024-51610-4 (PMC11344848; doi:10.1038/s41467-024-51610-4)
Supplement: Supplementary file 1 — Supplementary Information [file 41467_2024_51610_MOESM1_ESM.pdf]

SUPPLEMENTARY INFORMATION

Proximity-driven Site-specific Cyclization of Phage-displayed Peptides

Libby Brown,[1,3] Aldrin V. Vidal,[1] Ana Laura Dias[2], Tiago Rodrigues[2], Anna Sigurdardottir,[3] Toby Journeaux,[1] Siobhan O'Brien,[3] Thomas V. Murray,[3] Peter Ravn,[3,5] Monika Papworth,[3] Gonçalo J. L. Bernardes*[1,3]

[1] Yusuf Hamied Department of Chemistry, University of Cambridge, Lensfield Road, CB2 1EW Cambridge, UK.

[2] Instituto de Investigação do Medicamento (iMed), Faculdade de Farmácia, Universidade de Lisboa, Av. Prof. Gama Pinto, 1649-003 Lisboa, Portugal.

[3] Biologics Engineering, Oncology R&D, AstraZeneca, The Discovery Centre; Cambridge Biomedical Campus, 1 Francis Crick Avenue, CB2 0AACambridge, UK.

[4] Instituto de Medicina Molecular João Lobo Antunes, Faculdade de Medicina da Universidade de Lisboa, Av. Prof. Egas Moniz, 1649-028 Lisboa, Portugal.

[5] Present address: Department of Biotherapeutic Discovery, H. Lundbeck A/S, Valby, Denmark.

*e-mail: gb453@cam.ac.uk

Table of contents

1. Supplementary Figures 2
2. Supplementary Methods 13
3. Supplementary Tables 19
4. Supplementary References 23

1. Supplementary Figures

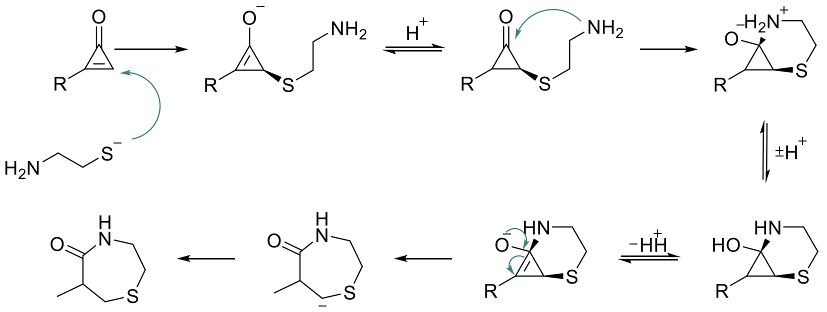

Supplementary Figure 1: Mechanism of CPO reaction with 1,2-amino thiols.

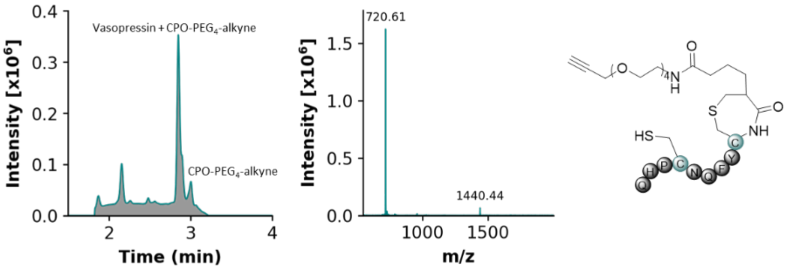

Supplementary Figure 2: HPLC and MS analysis of the reaction between peptide **3** – vasopressin (reduced) and CPO-PEG$_4$-alkyne (2 equiv.) in PBS (pH 7.4) after 2 h at 25 ºC.

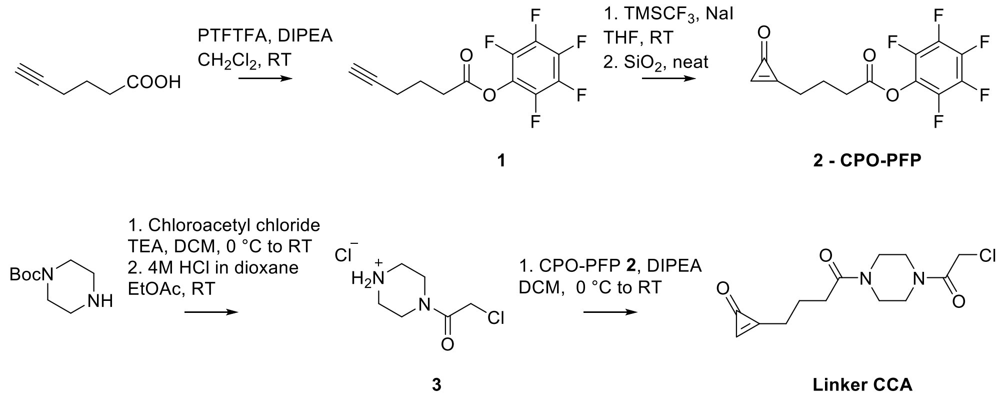

Supplementary Figure 3: Synthesis of linker CCA.

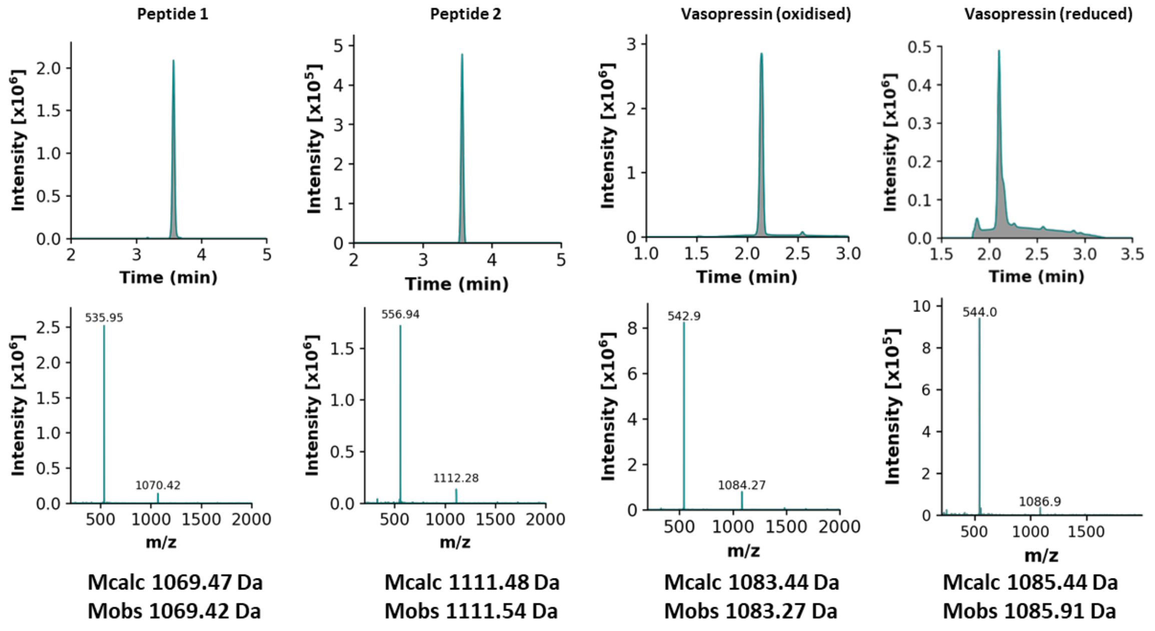

Peptide	AA sequiv.
1	**C**YFQNSPRG-NH_2
2	Ac-SYFQN**C**PRG-NH_2
3 (vasopressin)	**C**YFQN**C**PRG-NH_2

Supplementary Figure 4: HPLC and MS analysis of peptides **1**, **2** and **3** (vasopressin, oxidised and reduced).

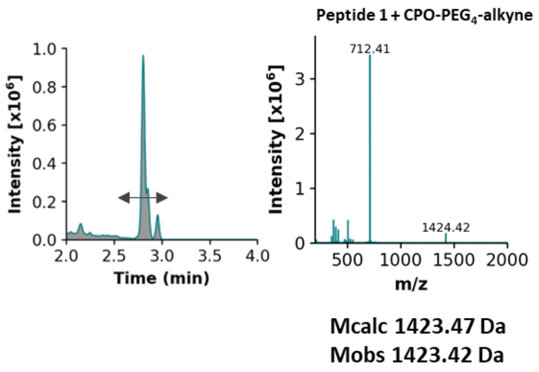

Supplementary Figure 5: HPLC and MS analysis of the reaction between peptide **1** and a 1:1 mix of CPO-PEG$_4$-alkyne: ClAc-piperazine (2 equiv. each) in PBS (pH 7.4) after 2 h at 25 ºC.

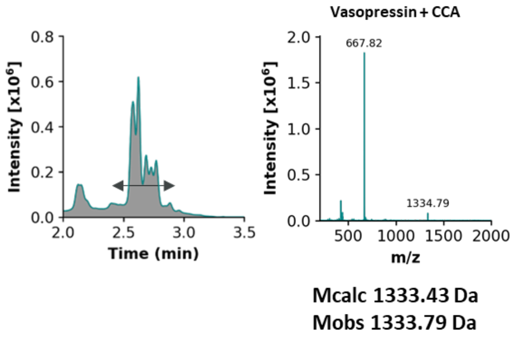

Supplementary Figure 6: HPLC and MS analysis of the reaction between peptide **3** – vasopressin (reduced) and linker CCA (2 equiv.) in PBS (pH 7.4) after 2 h at 25 °C.

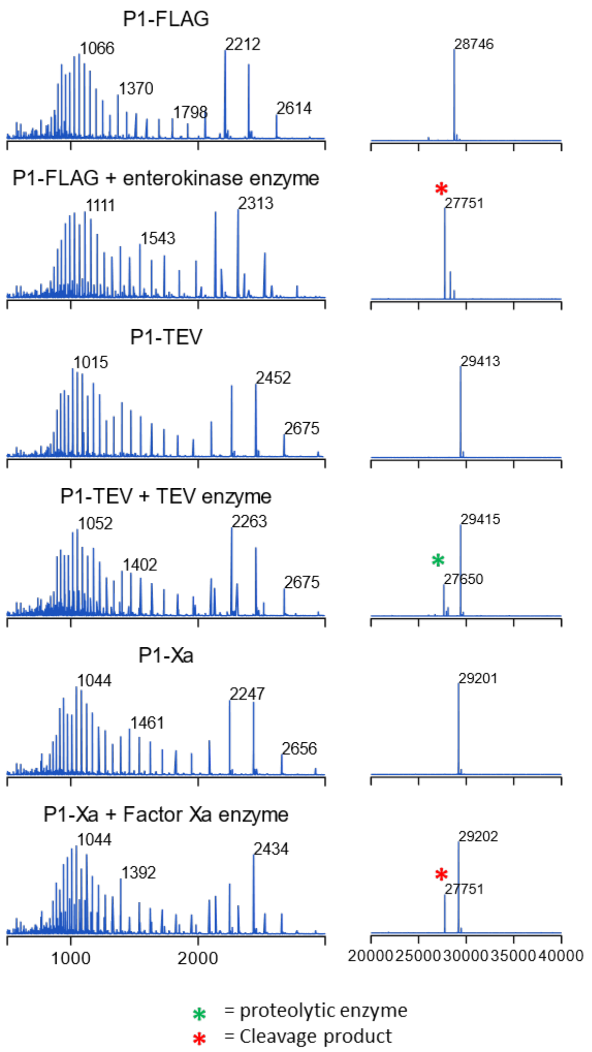

Supplementary Figure 7: Combined ion series and deconvoluted mass spectra of D1/D2-pep fusion proteins P1-FLAG, P1-TEV and P1-Xa before and after treatment with their respective proteolytic enzymes (O/N treatment at 25 °C). **P1-FLAG:** M_{calc} 28740 Da, M_{obs} 28746 Da. **P1-TEV:** M_{calc} 29407 Da, M_{obs} 29413 Da. **P1-Xa:** M_{calc} 29195 Da, M_{obs} 29201 Da. **P1:** M_{calc} 27741 Da, M_{obs} 27751.

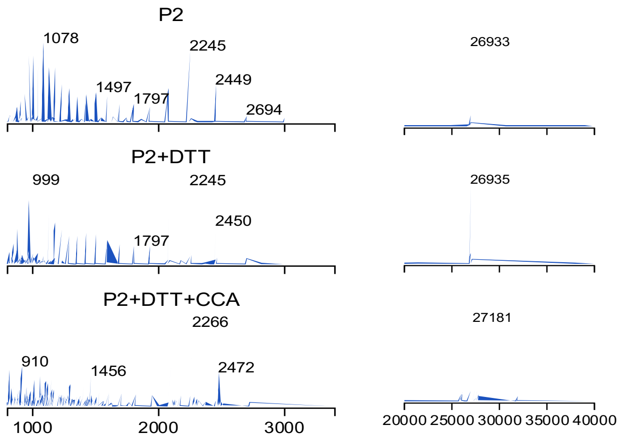

Supplementary Figure 8: Combined ion series and deconvoluted mass spectra of D1/D2-pep fusion proteins P2, P2 treated with DTT (100 equiv., 30 min, 37 °C) and P2 treated with DTT then CCA (25 equiv., 2 h, 25 ºC). **P2:** M_{calc} 26927 Da, M_{obs} 26933 Da. **P2 (reduced):** M_{calc} 26929 Da, M_{obs} 26935 Da. **P2-CCA:** M_{calc} 27175 Da, M_{obs} 27181 Da.

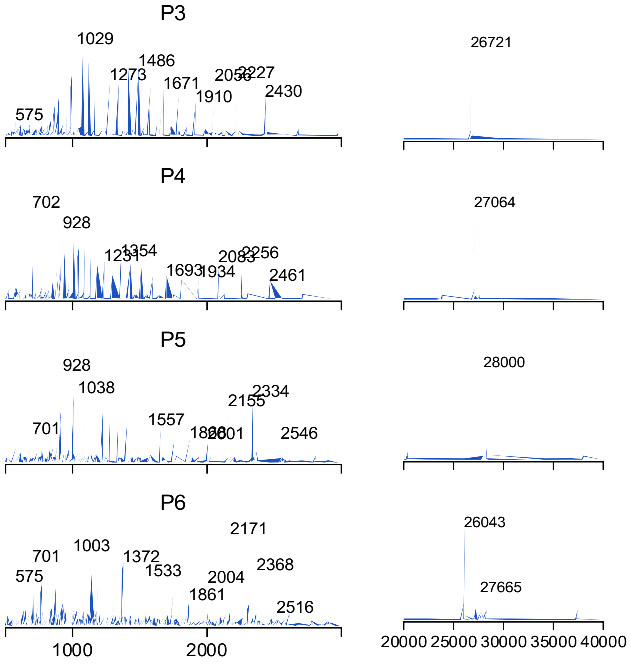

Supplementary Figure 9: Combined ion series and deconvoluted mass spectra of D1/D2-pep fusion proteins P3, P4, P5 and P6. **P3:** M_{calc} 26714 Da, M_{obs} 26721 Da. **P4:** M_{calc} 27058 Da, M_{obs} 27064 Da. **P5:** M_{calc} 27993 Da, M_{obs} 28000 Da. **P6:** M_{calc} 27657 Da, M_{obs} 27655 Da.

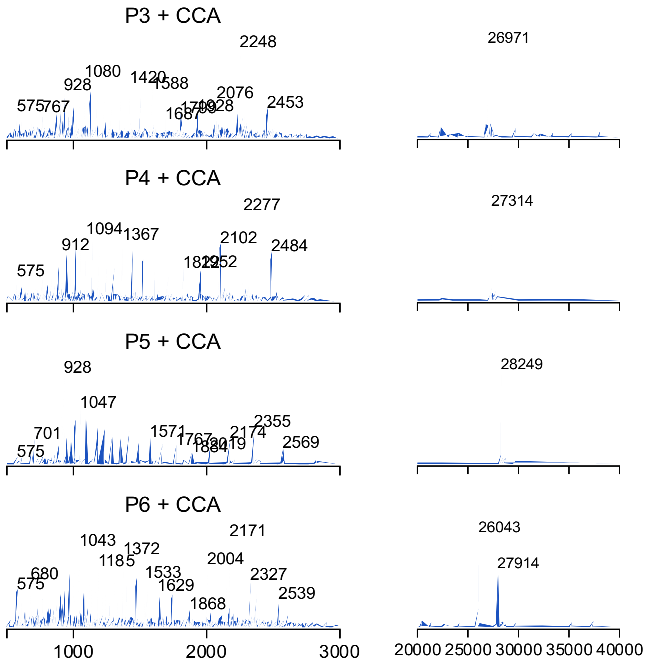

Supplementary Figure 10: Combined ion series and deconvoluted mass spectra of D1/D2-pep fusion proteins P3, P4, P5 and P6 treated with DTT (100 equiv., 30 min, 37 °C) then CCA (25 equiv., 2 h, 25 ºC). **P3-CCA:** M_{calc} 26963 Da, M_{obs} 26971 Da. **P4-CCA:** M_{calc} 27307 Da, M_{obs} 27314 Da. **P5-CCA:** M_{calc} 28242 Da, M_{obs} 28249 Da. **P6-CCA:** M_{calc} 27906 Da, M_{obs} 27914 Da.

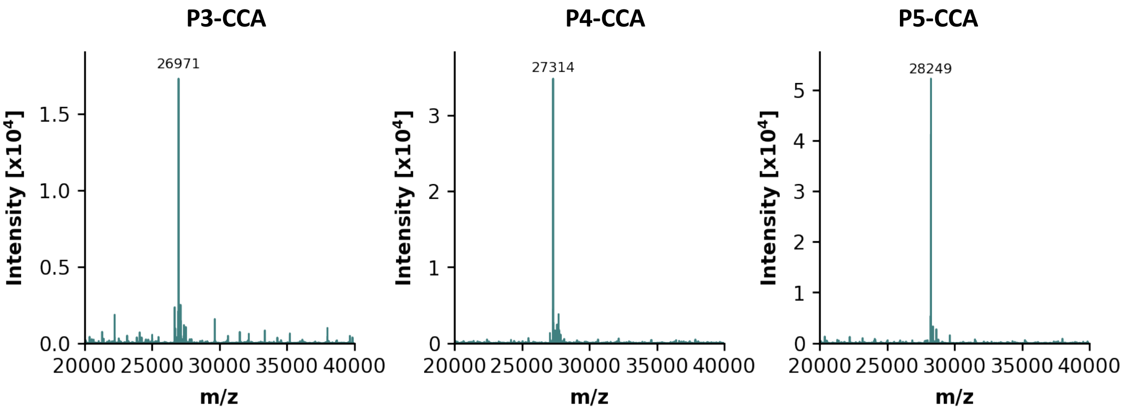

Supplementary Figure 11: LC-MS analysis of CCA-cyclized peptide-D1/D2 fusions P3, P4 and P5, following 24 h of incubation in a redox buffer containing 2 mM glutathione at 37 °C.

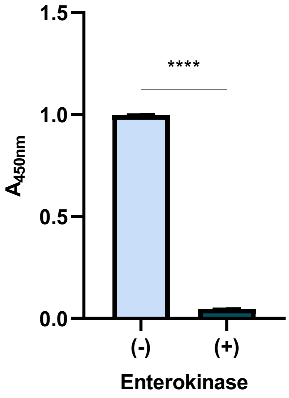

Supplementary Figure 12: Using phage ELISA to monitor the cleavage of a FLAG tag fused to the N-terminus of a phage-displayed peptide (96-well plated treated with anti-FLAG antibody, bound phage detected using anti-M13 HRP).

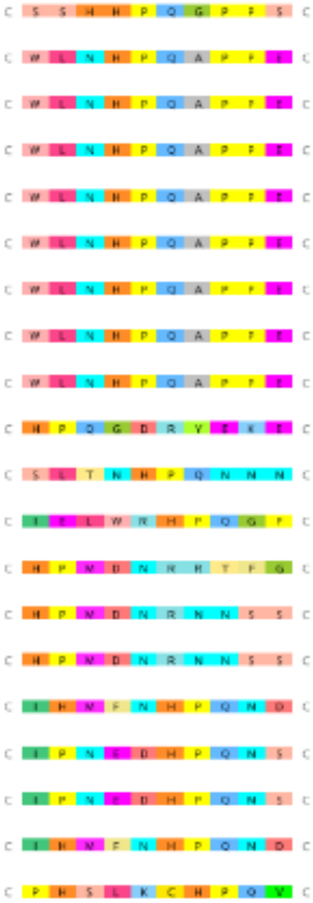

Supplementary Figure 13: Peptides isolated after three rounds of selection against streptavidin using library L1-Lin.

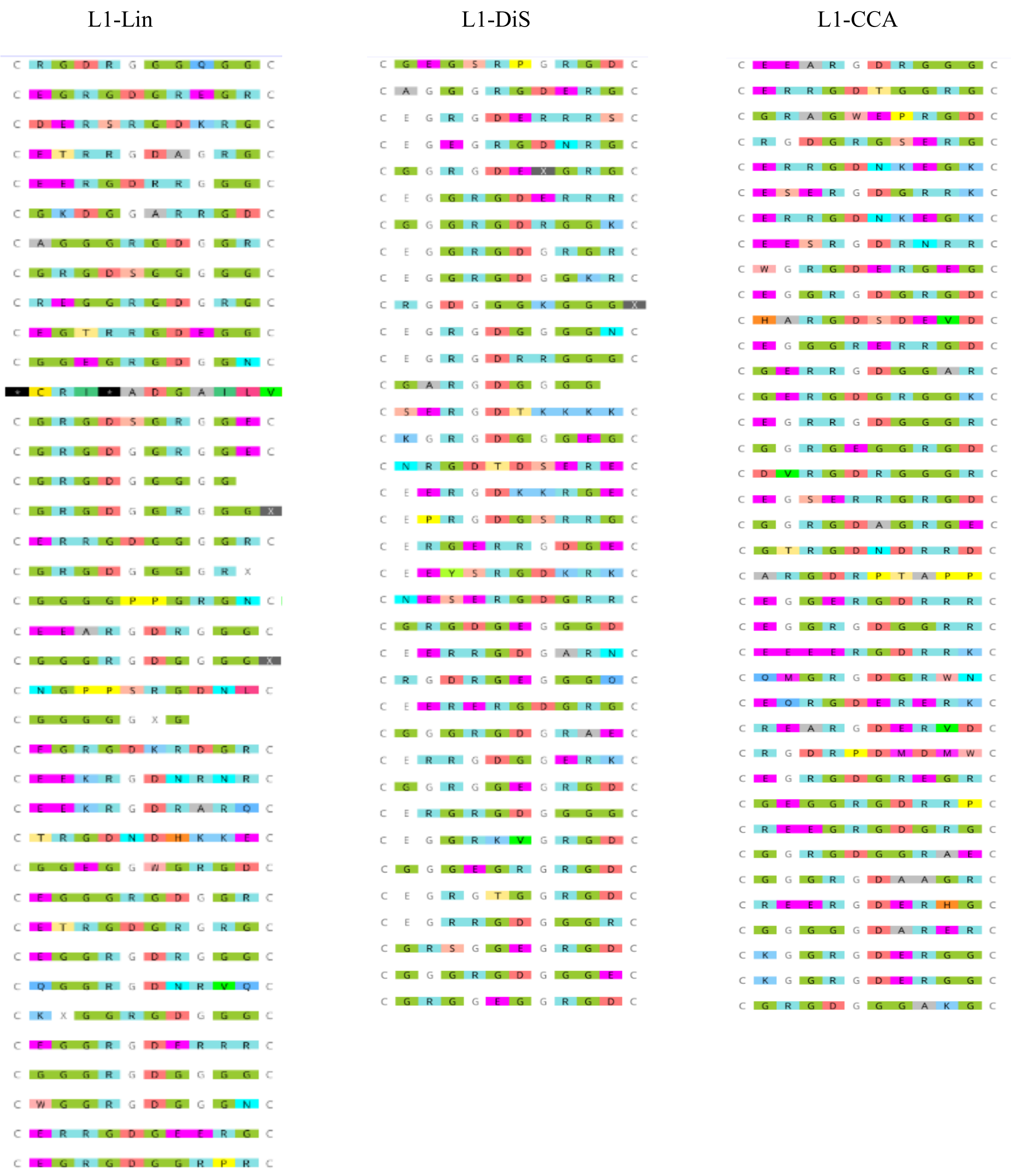

Supplementary Figure 14: Peptides isolated after three rounds of selection against αvβ3 using library L1-Lin, L1-DiS and L1-CCA.

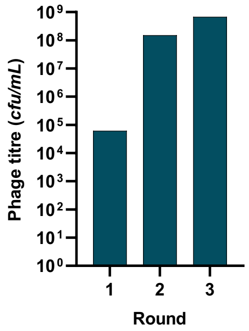

Supplementary Figure 15: Phage output titre values obtained for rounds **1**, **2** and **3** of selection against αvβ3 using library L1-CCA.

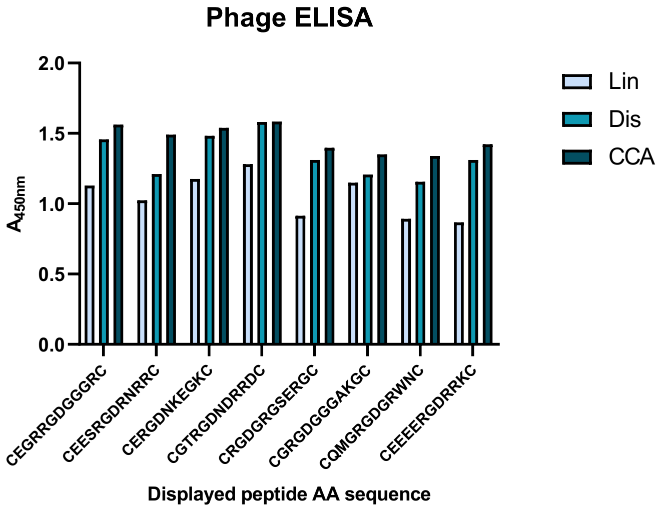

Supplementary Figure 16: Validation of the binding of peptides Av1-8 (linear, disulphide-bonded and CCA-cyclized) to αvβ3 using phage ELISA (bound phage detected using anti-M13-HRP).

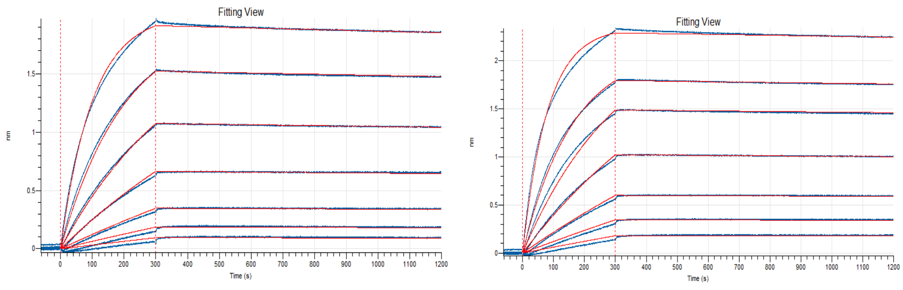

Av1 K_D = 14.1 ± 0.06 nM

Av2 K_D = 7.08 ± 0.08 nM

Supplementary Figure 17: Binding analysis of CCA cyclized peptides Av1 and Av2 (expressed as D1/D2 fusions) to αvβ3 using Bio-Layer Interferometry (BLI). The BLI sensorgrams show the real-time binding interactions between Av1 and Av2 peptides and

αvβ3. The binding responses, represented as changes in interference (nm), are shown as a function of time (seconds). $k_D = \frac{[A].[B]}{[AB]} = \frac{K_d}{K_a}$

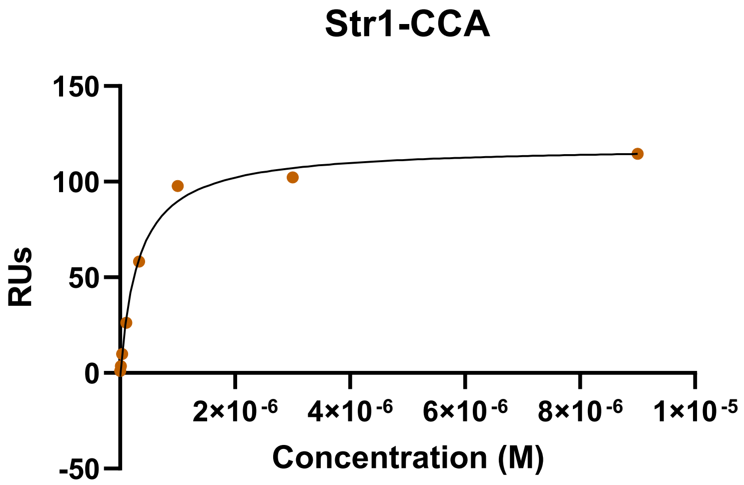

Supplementary Figure 18: Calculation of the binding affinity constant (Kd) of peptide Str1-CCA (analysis performed using Biacore 8K Evaluation Software). The data corresponds to one of three independent replicates. Experiments were performed in triplicate.

2. Supplementary Methods

Materials:

Unless otherwise stated, all materials and chemicals were purchased from Sigma-Aldrich, Invitrogen or Thermo Fisher Scientific and used with no further adjustment or purification. All restriction/ protease enzymes and ligation/ polymerase chain reaction (PCR)/ site-directed mutagenesis (SDM) master mixes were purchased from New England Biolabs (NEB). All primers and oligonucleotides were obtained from Sigma-Aldrich and string inserts from GeneArt (Thermo Fisher Scientific).

The following materials were prepared by AstraZeneca: 2xTYAG petri and bioassay plates, relevant antibiotics (ampicillin, kanamycin, chloramphenicol), 20% (v/v) glucose solution, 50% (v/v) glycerol solution and materials for protein expression and purification, Kunkel mutagenesis, phage production and precipitation, and phage display selections.

The plasmid maps and full-length protein sequences associated with AstraZeneca cannot be shared or discussed due to proprietary reasons.

i. Antibiotics

	**Diluent**	**Final concentration**
Ampicillin	Distilled water	100 mg/mL
Kanamycin	Distilled water	50 mg/mL
Chloramphenicol	50% (v/v) ethanol in distilled water	10 mg/mL

ii. Media and buffers

Media and buffers were prepared by AstraZeneca unless otherwise stated.

2xTY media (1 L)	Bacto-tryptone (16 g), Bacto Yeast Extract (10 g), NaCl (5 g). Adjust to pH 7.0 with NaOH. Add distilled water to a total volume of 1 L.
2xTYAG	2xTY, ampicillin (100 ug/mL), glucose (2% v/v)
2xTYAK	2xTY, ampicillin (100 ug/mL), kanamycin (50 ug/mL)
2xTYAGC	2xTY, ampicillin (100 ug/mL), glucose (2% v/v), chloramphenicol (10 ug/mL)
PBS	Supplied by Gibco. Manufactured without calcium, magnesium or phenol red.
3% mPBS	PBS with 3% (w/v) powdered milk

PBST	PBS with 0.1% (v/v) Tween-20
TE	10 mM Tris-HCl pH 8.0, 0.1mM EDTA
Aspec A	50 mM Tris-HCl pH 8.0, 300 mM NaCl
Aspec C	50 mM Tris-HCl pH 8.0, 300 mM NaCl, 400 mM imidazole
TES	200 mM Tris-HCl pH 8.0, 0.5 mM EDTA, 0.5 M sucrose
TAE	40 mM Tris-HCl pH 8.0, 20 mM acetate, 1 mM EDTA

iii. Competent cells

	Supplier	Genotype
Mix and go! Dh5-α	Zymo	F-Φ80lacZΔM15 Δ(lacZYA-argF)U169 deoR nupG recA1 endA1 hsdR17(rK- mK+) phoA glnV44 (supE44) thi-1 gyrA96 relA1,λ-
TG1	AstraZeneca	F'[traD36 lacIq Δ(lacZ) M15 proA+B+] glnV (supE) thi-1 Δ(mcrB-hsdSM)5 (rK- mK- McrB-) thi Δ(lac-proAB)
CJ236	AstraZeneca	[F' Tra+ Pil+ (CamR)] ung-1 relA1 dut-1 thi-1 spoT1 mcrA
BL21 (DE3)	NEB	fhuA2 [lon] ompT gal (λ DE3) [dcm] ∆hsdS<br>λ DE3 = λ sBamHIo ∆EcoRI-B int::(lacI::PlacUV5::T7 gene1) i21 ∆nin5

iv. List of strings

P1-FLAG:

CAGGAAACAGCTATGACCATGATTACGCCAAGCTTTGGAGCCTTTTTTTTGGAGATTTTC
AACGTGAAAAAATTATTATTCGCAATTCCTTTAGTTGTTCCTTTCTATGCGGCCCAGCCG
GCCATGGCCGACTACAAAGACGATGACGACAAGTGTACCACTGAAGAGCCTTATTTAGT
TTCTTTGTGTTGGTTAGCGGCCGCAGGTGGTTCTGGTTCTACCGCTGG

P1-TEV:

CAGGAAACAGCTATGACCATGATTACGCCAAGCTTTGGAGCCTTTTTTTTGGAGATTTTC
AACGTGAAAAAATTATTATTCGCAATTCCTTTAGTTGTTCCTTTCTATGCGGCCCAGCCG
GCCATGGCCGACTACAAAGACGATGACGACAAGGAAAATCTATACTTTTGTACCACTGA
AGAGCCTTATTTAGTTTCTTTGTGTTGGTTAGCGGCCGCAGGTGGTTCTGGTTCTACCG
CTGG

P1-Xa:

CAGGAAACAGCTATGACCATGATTACGCCAAGCTTTGGAGCCTTTTTTTTGGAGATTTTC
AACGTGAAAAAATTATTATTCGCAATTCCTTTAGTTGTTCCTTTCTATGCGGCCCAGCCG
GCCATGGCCGACTACAAAGACGATGACGACAAGATTGAAGGTCGTTGTACCACTGAAG
AGCCTTATTTAGTTTCTTTGTGTTGGTTAGCGGCCGCAGGTGGTTCTGGTTCTACCGCT
GG

P2-FLAG:

CAGGAAACAGCTATGACCATGATTACGCCAAGCTTTGGAGCCTTTTTTTTGGAGATTTTC
AACGTGAAAAAATTATTATTCGCAATTCCTTTAGTTGTTCCTTTCTATGCGGCCCAGCCG
GCCATGGCCGACTACAAAGACGATGACGACAAGTGTGGAGGTAGCGGAGGTTGTTGG
TTAGCGGCCGCAGGTGGTTCTGGTTCTACCGCTGG

P3-FLAG:

CAGGAAACAGCTATGACCATGATTACGCCAAGCTTTGGAGCCTTTTTTTTGGAGATTTTC
AACGTGAAAAAATTATTATTCGCAATTCCTTTAGTTGTTCCTTTCTATGCGGCCCAGCCG
GCCATGGCCGACTACAAAGACGATGACGACAAGTGTGGAAGCGGTGGAAGCGGATGT
GCGGCCGCAGGTGGTTCTGGTTCTACCGCTGG

P4-FLAG:

CAGGAAACAGCTATGACCATGATTACGCCAAGCTTTGGAGCCTTTTTTTTGGAGATTTTC
AACGTGAAAAAATTATTATTCGCAATTCCTTTAGTTGTTCCTTTCTATGCGGCCCAGCCG
GCCATGGCCGACTACAAAGACGATGACGACAAGTGTCAGCCGCACCCGGGACAGACC
TGTGCGGCCGCAGGTGGTTCTGGTTCTACCGCTGG

v. List of oligonucleotides:

L1:

CCCGTGATGGTGATGATGATGTGCGGCCGCACCACCGCASNNSNNSNNSNNSNNSNN
SNNSNNSNNSNNACAGGCCATGGCCGGCTGGGCCGCATAGAAAGG

vi. List of primers:

Str3_Fwd	ggccccccctctactgcGCGGCCGCAGGTGGAGGC	Str3_Rev	ggcgatgttgtagggacaGGCCATGGCCGGCTGGGC
Str4_Fwd	gtccccttcatcgactgcGCGGCCGCAGGTGGAGGC	Str4_Rev	gaggatgttgaagggacaGGCCATGGCCGGCTGGGC
Str5_Fwd	aaccccaccttcgactgcGCGGCCGCAGGTGGAGGC	Str5_Rev	gatgatgttgtagggacaGGCCATGGCCGGCTGGGC
Str6_Fwd	tccccсttctacggctgcGCGGCCGCAGGTGGAGGC	Str6_Rev	ggcgatgttgaacggacaGGCCATGGCCGGCTGGGC
Str7_Fwd	tacagcaccggcacctgcGCGGCCGCAGGTGGAGGC	Str7_Rev	ctggacgttgatgggacaGGCCATGGCCGGCTGGGC
Str8_Fwd	cgccccgactcccactgcGCGGCCGCAGGTGGAGGC	Str8_Rev	ctcgacgttgtagggacaGGCCATGGCCGGCTGGGC
StrPC_Fwd	caggccccgcccgagtgcGCGGCCGCAGGTGGAGGC	StrPC_Rev	ggggtggttgagccaacaGGCCATGGCCGGCTGGGC
avb3-1_Fwd	ggtggtggccgttgcggtggcGCGGCCGCAGGTGGAGGC	avb3-1_Rev	atcaccacgacgaccttcacaGGCCATGGCCGGCTGGGC
avb3-2_Fwd	cgtaatcgccgttgcggcggcGCGGCCGCAGGTGGAGGC	avb3-2_Rev	atcgccacggctttcttcacaGGCCATGGCCGGCTGGGC
avb3-3_Fwd	agaaggcaaatgcggcggcGCGGCCGCAGGTGGAGGC	avb3-3_Rev	ttgttatcgccgcgttcgcaGGCCATGGCCGGCTGGGC
avb3-4_Fwd	gatcgtcgtgattgcggcggcGCGGCCGCAGGTGGAGGC	avb3-4_Rev	attatcgccacgggtgccacaGGCCATGGCCGGCTGGGC
avb3-5_Fwd	agcgaacgtggttgcggaggcGCGGCCGCAGGTGGAGGC	avb3-5_Rev	accacggccatcaccacgacaGGCCATGGCCGGCTGGGC
avb3-6_Fwd	ggtgcgaaaggttgcggtggcGCGGCCGCAGGTGGAGGC	avb3-6_Rev	accaccatcgccacggccacaGGCCATGGCCGGCTGGGC
avb3-7_Fwd	ggccgttggaattgtggcggtGCGGCCGCAGGTGGAGGC	avb3-7_Rev	atcgccacggcccatctgacaGGCCATGGCCGGCTGGGC
avb3-8_Fwd	gatcgtcgtaaatgcggcggcGCGGCCGCAGGTGGAGGC	avb3-8_Rev	gccgcgttcttcttcttcgcaGGCCATGGCCGGCTGGGC

vii. Peptides purchased from Genscript:

Peptide	AA sequiv.	Mass (Da)
1	**C**YFQNSPRG-NH_2	1069.42
2	Ac-SYFQN**C**PRG-NH_2	1111.28
3	**C**YFQN**C**PRG-NH_2	1083.27 (cysteines oxidised)

i. Stop template ST1 used for the preparation of dU-ssDNA:

Stop template	Displayed peptide (AA sequiv.)	Displayed peptide (nucleotide sequiv.)
ST1	*CRI*ADGAILVCG	TGATGCCGTATTTGAGCTGATGGTGCTATTCTGGTGTGCGGC

Chemical Synthesis:

The synthesis of CPO-PFP and CPO-PEG$_2$-biotin were achieved following a protocol previously reported by our group.[1]

i. 2-chloro-1-(piperazin-1-yl)ethan-1-one (1)

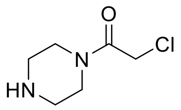

To a stirring solution of 1-Boc-piperazine (1000 mg, 1 eq, 5.37 mmol) and DIPEA (833 mg, 1.12 mL, 1.2 eq, 6.44 mmol) in 3 mL dry DCM at 0 ºC was added dropwise a 5 mL solution of 2-chloroacetyl chloride (728 mg, 512 µL, 1.2 eq, 6.44 mmol) in DCM. The reaction was stirred for 2 h at room temperature. After that time, the reaction was diluted with another 50 mL DCM and washed with two portions of 25 mL water. The organic phase was dried over magnesium sulphate and concentrated in vacuo to afford a dark brown oil bearing 93% purity (UHPLC-UV trace) of the desired compound. R_f. DCM/MeOH 10:1= 0.66

Quantitative *N*-Boc deprotection was performed by dissolving the compound in 10 mL TFA/DCM 4:6 and sitting for 2 h at room temperature. R_f of deprotected product in DCM/MeOH 10:1= 0.14. The resulting compound was utilized in the next synthetic step without further purification.

ii. 2-(4-(4-(2-Chloroacetyl)piperazin-1-yl)-4-oxobutyl)cycloprop-2-en-1-one (Linker CCA)

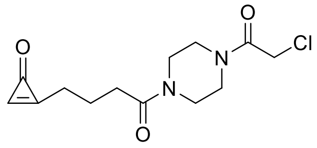

CPO-PFP (85 mg, 1 eq, 0.28 mmol) and DIPEA (92µL, 68 mg, 1.9 eq, 0.53 mmol) were dissolved in 3 mL dry DCM and stirred for 2 min at 0 ºC. Then a solution of 2-chloro-1-(piperazin-1-yl)ethan-1-one (28 mg, 0.75 Eq, 0.21 mmol) in 5 mL DCM was slowly added. After 2 h, the reaction was concentrated. The crude product was purified by column chromatography starting with a 30:1 DCM/MeOH gradient to 10:1 (R_f DCM/MeOH 10:1 = 0.67) to afford Linker CCA as pale yellow oil (21 mg, 35% yield).

^{1}H NMR (500 MHz, $CDCl_3$) δ = 8.45 (s, 1H), 4.07 (s, 2H), 3.70-3.49 (m, 8H), 2.81-2.78 (m, 2H), 2.55-2.50 (m, 2H), 2.06 (p, J = 7.1 Hz, 2H).

^{13}C NMR (126 MHz, $CDCl_3$) δ = 170.40 (C), 170.24 (C), 169.65 (C), 165.53 (C), 148.49 (CH), 46.26 (CH_2), 46.04 (CH_2), 40.79 (CH_2), 31.58 (CH_2), 27.24 (CH_2), 21.04 (CH_2).

ESI^+-HRMS: calculated for $C_{13}H_{17}ClN_2O_3$ ($[M+H]^+$) 285.1006, found 285.1129.

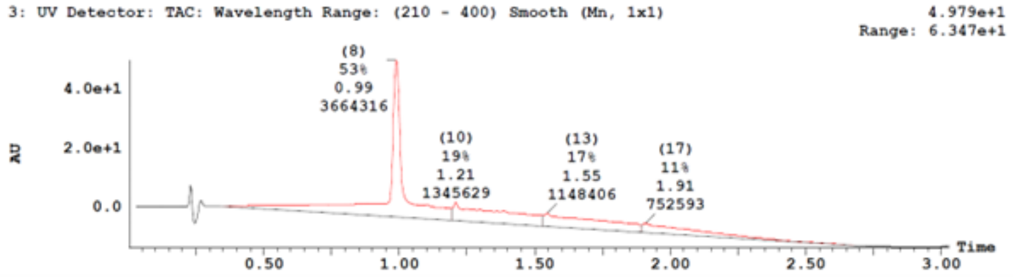

Supplementary Figure 19: Linker CCA UHPLC (UV trace)

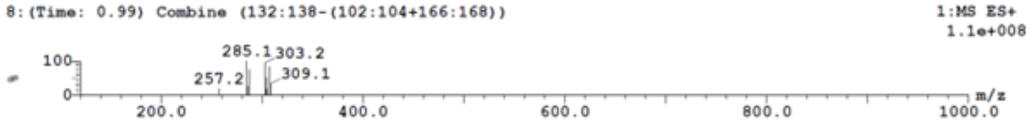

Supplementary Figure 20: Linker CCA ESI+MS

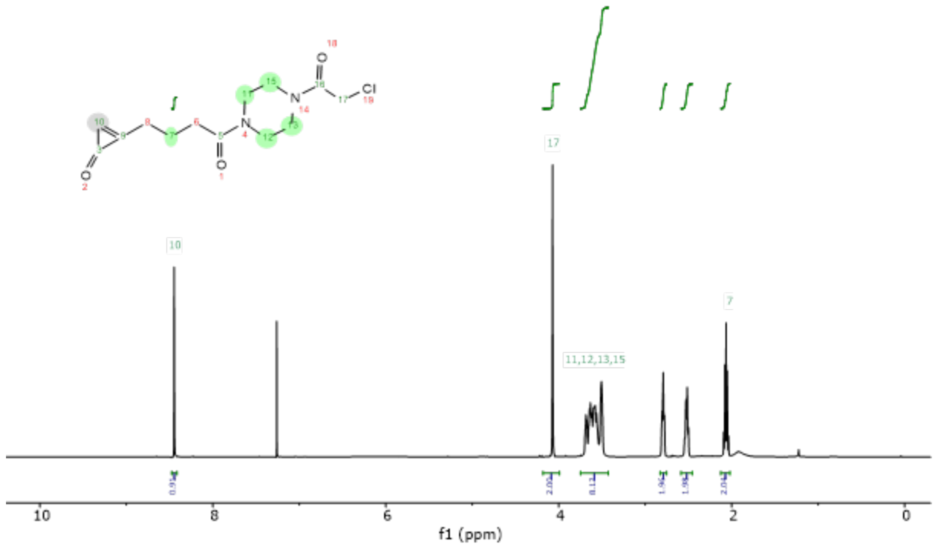

Supplementary Figure 21: Linker CCA ^{1}H-NMR

3. Supplementary Tables

Supplementary Table 1: Peptide sequences for linear peptides, HPQ1-12

Peptide name	Peptide sequence	Peptide sequence SMILES	Peptide sequence SMILES positional	Peptide sequence text
HPQ1	CWLNHPQGPPSC	N[C@@]([H])(CS)C(=O)N[C@@]([H])(CC(=CN2)C1=C2C=CC=C1)C(=O)N[C@@]([H])(CC(C)C)C(=O)N[C@@]([H])(CC(=O)N)C(=O)N[C@@]([H])(CC1=CN=C-N1)C(=O)N1[C@@]([H])(CCC1)C(=O)N[C@@]([H])(CCC(=O)N)C(=O)NCC(=O)N1[C@@]([H])(CCC1)C(=O)N1[C@@]([H])(CCC1)C(=O)N[C@@]([H])(CO)C(=O)N[C@@]([H])(CS)C(=O)O	['C(C(C(=O)O)N)S', 'C1=CC=C2C(=C1)C(=CN2)CC(C(=O)O)N', 'CC(C)CC(C(=O)O)N', 'C(C(C(=O)O)N)C(=O)N', 'C1=C(NC=N1)C(C(=O)O)N', 'C1CC(NC1)C(=O)O', 'C(CC(=O)N)(C(=O)O)N', 'C(C(=O)O)N', 'C1CC(NC1)C(=O)O', 'C1CC(NC1)C(=O)O', 'C(C(C(=O)O)N)O', 'C(C(C(=O)O)N)S']	'cysteine tryptophan leucine asparagine histidine proline glutamine glycine proline proline serine cysteine'
HPQ2	CWLNHPQAPPEC	N[C@@]([H])(CS)C(=O)N[C@@]([H])(CC(=CN2)C1=C2C=CC=C1)C(=O)N[C@@]([H])(CC(C)C)C(=O)N[C@@]([H])(CC(=O)N)C(=O)N[C@@]([H])(CC1=CN=C-N1)C(=O)N1[C@@]([H])(CCC1)C(=O)N[C@@]([H])(CCC(=O)N)C(=O)N[C@@]([H])(C)C(=O)N1[C@@]([H])(CCC1)C(=O)N1[C@@]([H])(CCC1)C(=O)N[C@@]([H])(CCC(=O)O)C(=O)N[C@@]([H])(CS)C(=O)O	['C(C(C(=O)O)N)S', 'C1=CC=C2C(=C1)C(=CN2)CC(C(=O)O)N', 'CC(C)CC(C(=O)O)N', 'C(C(C(=O)O)N)C(=O)N', 'C1=C(NC=N1)C(C(=O)O)N', 'C1CC(NC1)C(=O)O', 'C(CC(=O)N)(C(=O)O)N', 'CC(C(=O)O)N', 'C1CC(NC1)C(=O)O', 'C1CC(NC1)C(=O)O', 'C(CC(=O)O)(C(=O)O)N', 'C(C(C(=O)O)N)S']	'cysteine tryptophan leucine asparagine histidine proline glutamine alanine proline proline glutamate cysteine'
HPQ3	CHPQGDRYEKEC	N[C@@]([H])(CS)C(=O)N[C@@]([H])(CC1=CN=C-N1)C(=O)N1[C@@]([H])(CCC1)C(=O)N[C@@]([H])(CCC(=O)N)C(=O)NCC(=O)N[C@@]([H])(CC(=O)O)C(=O)N[C@@]([H])(CCCNC(=N)N)C(=O)N[C@@]([H])(Cc1ccc(O)cc1)C(=O)N[C@@]([H])(CCC(=O)O)C(=O)N[C@@]([H])(CCCCN)C(=O)N[C@@]([H])(CCC(=O)O)C(=O)N[C@@]([H])(CS)C(=O)O	['C(C(C(=O)O)N)S', 'C1=C(NC=N1)C(C(=O)O)N', 'C1CC(NC1)C(=O)O', 'C(CC(=O)N)(C(=O)O)N', 'C(C(=O)O)N', 'C(C(C(=O)O)N)C(=O)O', 'C(C(C(=O)O)N)CN=C(N)N', 'C1=CC(=CC=C1CC(C(=O)O)N)O', 'C(CC(=O)O)(C(=O)O)N', 'C(CCN)CC(C(=O)O)N', 'C(CC(=O)O)(C(=O)O)N', 'C(C(C(=O)O)N)S']	'cysteine histidine proline glutamine glycine aspartate arginine tyrosine glutamate lysine glutamate cysteine'
HPQ4	CSLTNHPQNFNC	N[C@@]([H])(CS)C(=O)N[C@@]([H])(CO)C(=O)N[C@@]([H])(CC(C)C)C(=O)N[C@@]([H])([C@]([H])(O)C)C(=O)N[C@@]([H])(CC(=O)N)C(=O)N[C@@]([H])(CC1=CN=C-N1)C(=O)N1[C@@]([H])(CCC1)C(=O)N[C@@]([H])(CCC(=O)N)C(=O)N[C@@]([H])(CC(=O)N)C(=O)N[C@@]([H])(Cc1ccccc1)C(=O)N[C@@]([H])(CC(=O)N)C(=O)N[C@@]([H])(CS)C(=O)O	['C(C(C(=O)O)N)S', 'C(C(C(=O)O)N)O', 'CC(C)CC(C(=O)O)N', 'C(C(C(=O)O)N)(O)C', 'C(C(C(=O)O)N)C(=O)N', 'C1=C(NC=N1)C(C(=O)O)N', 'C1CC(NC1)C(=O)O', 'C(CC(=O)N)(C(=O)O)N', 'C(C(C(=O)O)N)C(=O)N', 'C1=CC=C(C=C1)CC(C(=O)O)N', 'C(C(C(=O)O)N)C(=O)N', 'C(C(C(=O)O)N)S']	'cysteine serine leucine threonine asparagine histidine proline glutamine asparagine phenylalanine asparagine cysteine'
HPQ5	CIELWRHPQGPC	N[C@@]([H])(CS)C(=O)N[C@@]([H])([C@]([H])(CC)C)C(=O)N[C@@]([H])(CCC(=O)O)C(=O)N[C@@]([H])(CC(C)C)C(=O)N[C@@]([H])(CC(=CN2)C1=C2C=CC=C1)C(=O)N[C@@]([H])(CCCNC(=N)N)C(=O)N[C@@]([H])(CC1=CN=C-N1)C(=O)N1[C@@]([H])(CCC1)C(=O)N[C@@]([H])(CCC(=O)N)C(=O)NCC(=O)N1[C@@]([H])(CCC1)C(=O)N[C@@]([H])(CS)C(=O)O	['C(C(C(=O)O)N)S', 'CCC(C)C(C(=O)O)N', 'C(CC(=O)O)(C(=O)O)N', 'CC(C)CC(C(=O)O)N', 'C1=CC=C2C(=C1)C(=CN2)CC(C(=O)O)N', 'C(C(C(=O)O)N)CN=C(N)N', 'C1=C(NC=N1)C(C(=O)O)N', 'C1CC(NC1)C(=O)O', 'C(CC(=O)N)(C(=O)O)N', 'C(C(=O)O)N', 'C1CC(NC1)C(=O)O', 'C(C(C(=O)O)N)S']	'cysteine isoleucine glutamate leucine tryptophan arginine histidine proline glutamine glycine proline cysteine'
HPQ6	CHPQDNRRTFGC	N[C@@]([H])(CS)C(=O)N[C@@]([H])(CC1=CN=C-N1)C(=O)N1[C@@]([H])(CCC1)C(=O)N[C@@]([H])(CCC(=O)N)C(=O)N[C@@]([H])(CC(=O)O)C(=O)N[C@@]([H])(CC(=O)N)C(=O)N[C@@]([H])(CCCNC(=N)N)C(=O)N[C@@]([H])(CCCNC(=N)N)C(=O)N[C@@]([H])([C@]([H])(O)C)C(=O)N[C@@]([H])(Cc1ccccc1)C(=O)NCC(=O)N[C@@]([H])(CS)C(=O)O	['C(C(C(=O)O)N)S', 'C1=C(NC=N1)C(C(=O)O)N', 'C1CC(NC1)C(=O)O', 'C(CC(=O)N)(C(=O)O)N', 'C(C(C(=O)O)N)C(=O)O', 'C(C(C(=O)O)N)C(=O)N', 'C(C(C(=O)O)N)CN=C(N)N', 'C(C(C(=O)O)N)CN=C(N)N', 'C(C(C(=O)O)N)(O)C', 'C1=CC=C(C=C1)CC(C(=O)O)N', 'C(C(=O)O)N', 'C(C(C(=O)O)N)S']	'cysteine histidine proline glutamine aspartate asparagine arginine arginine threonine phenylalanine glycine cysteine'

HPQ7	CHPQDNRNNSSC	N[C@@]([H])(CS)C(=O)N[C@@]([H])(CC1=CN=C-N1)C(=O)N1[C@@]([H])(CCC1)C(=O)N[C@@]([H])(CCC(=O)N)C(=O)N[C@@]([H])(CC(=O)O)C(=O)N[C@@]([H])(CC(=O)N)C(=O)N[C@@]([H])(CCCNC(=N)N)C(=O)N[C@@]([H])(CC(=O)N)C(=O)N[C@@]([H])(CC(=O)N)C(=O)N[C@@]([H])(CO)C(=O)N[C@@]([H])(CO)C(=O)N[C@@]([H])(CS)C(=O)O	['C(C(C(=O)O)N)S', 'C1=C(NC=N1)C(C(=O)O)N', 'C1CC(NC1)C(=O)O', 'C(CC(=O)N)(C(=O)O)N', 'C(C(C(=O)O)N)C(=O)O', 'C(C(C(=O)O)N)C(=O)N', 'C(C(C(=O)O)N)CN=C(N)N', 'C(C(C(=O)O)N)C(=O)N', 'C(C(C(=O)O)N)C(=O)N', 'C(C(C(=O)O)N)O', 'C(C(C(=O)O)N)O', 'C(C(C(=O)O)N)S']	'cysteine histidine proline glutamine aspartate asparagine arginine asparagine asparagine serine serine cysteine'
HPQ8	CHPQDNRANSSC	N[C@@]([H])(CS)C(=O)N[C@@]([H])(CC1=CN=C-N1)C(=O)N1[C@@]([H])(CCC1)C(=O)N[C@@]([H])(CCC(=O)N)C(=O)N[C@@]([H])(CC(=O)O)C(=O)N[C@@]([H])(CC(=O)N)C(=O)N[C@@]([H])(CCCNC(=N)N)C(=O)N[C@@]([H])(C)C(=O)N[C@@]([H])(CC(=O)N)C(=O)N[C@@]([H])(CO)C(=O)N[C@@]([H])(CO)C(=O)N[C@@]([H])(CS)C(=O)O	['C(C(C(=O)O)N)S', 'C1=C(NC=N1)C(C(=O)O)N', 'C1CC(NC1)C(=O)O', 'C(CC(=O)N)(C(=O)O)N', 'C(C(C(=O)O)N)C(=O)O', 'C(C(C(=O)O)N)C(=O)N', 'C(C(C(=O)O)N)CN=C(N)N', 'CC(C(=O)O)N', 'C(C(C(=O)O)N)C(=O)N', 'C(C(C(=O)O)N)O', 'C(C(C(=O)O)N)O', 'C(C(C(=O)O)N)S']	'cysteine histidine proline glutamine aspartate asparagine arginine alanine asparagine serine serine cysteine'
HPQ9	CIHMFNHPQNDC	N[C@@]([H])(CS)C(=O)N[C@@]([H])([C@]([H])(CC)C)C(=O)N[C@@]([H])(CC1=CN=C-N1)C(=O)N[C@@]([H])(CCSC)C(=O)N[C@@]([H])(Cc1ccccc1)C(=O)N[C@@]([H])(CC(=O)N)C(=O)N[C@@]([H])(CC1=CN=C-N1)C(=O)N1[C@@]([H])(CCC1)C(=O)N[C@@]([H])(CCC(=O)N)C(=O)N[C@@]([H])(CC(=O)N)C(=O)N[C@@]([H])(CC(=O)O)C(=O)N[C@@]([H])(CS)C(=O)O	['C(C(C(=O)O)N)S', 'CCC(C)C(C(=O)O)N', 'C1=C(NC=N1)C(C(=O)O)N', 'CSCCC(C(=O)O)N', 'C1=CC=C(C=C1)CC(C(=O)O)N', 'C(C(C(=O)O)N)C(=O)N', 'C1=C(NC=N1)C(C(=O)O)N', 'C1CC(NC1)C(=O)O', 'C(CC(=O)N)(C(=O)O)N', 'C(C(C(=O)O)N)C(=O)N', 'C(C(C(=O)O)N)C(=O)O', 'C(C(C(=O)O)N)S']	'cysteine isoleucine histidine methionine phenylalanine asparagine histidine proline glutamine asparagine aspartate cysteine'
HPQ10	CIPNEDHPQNSC	N[C@@]([H])(CS)C(=O)N[C@@]([H])([C@]([H])(CC)C)C(=O)N1[C@@]([H])(CCC1)C(=O)N[C@@]([H])(CC(=O)N)C(=O)N[C@@]([H])(CCC(=O)O)C(=O)N[C@@]([H])(CC(=O)O)C(=O)N[C@@]([H])(CC1=CN=C-N1)C(=O)N1[C@@]([H])(CCC1)C(=O)N[C@@]([H])(CCC(=O)N)C(=O)N[C@@]([H])(CC(=O)N)C(=O)N[C@@]([H])(CO)C(=O)N[C@@]([H])(CS)C(=O)O	['C(C(C(=O)O)N)S', 'CCC(C)C(C(=O)O)N', 'C1CC(NC1)C(=O)O', 'C(C(C(=O)O)N)C(=O)N', 'C(CC(=O)O)(C(=O)O)N', 'C(C(C(=O)O)N)C(=O)O', 'C1=C(NC=N1)C(C(=O)O)N', 'C1CC(NC1)C(=O)O', 'C(CC(=O)N)(C(=O)O)N', 'C(C(C(=O)O)N)C(=O)N', 'C(C(C(=O)O)N)O', 'C(C(C(=O)O)N)S']	'cysteine isoleucine proline asparagine glutamate aspartate histidine proline glutamine asparagine serine cysteine'
HPQ11	CIHMFNHPQNDC	N[C@@]([H])(CS)C(=O)N[C@@]([H])([C@]([H])(CC)C)C(=O)N[C@@]([H])(CC1=CN=C-N1)C(=O)N[C@@]([H])(CCSC)C(=O)N[C@@]([H])(Cc1ccccc1)C(=O)N[C@@]([H])(CC(=O)N)C(=O)N[C@@]([H])(CC1=CN=C-N1)C(=O)N1[C@@]([H])(CCC1)C(=O)N[C@@]([H])(CCC(=O)N)C(=O)N[C@@]([H])(CC(=O)N)C(=O)N[C@@]([H])(CC(=O)O)C(=O)N[C@@]([H])(CS)C(=O)O	['C(C(C(=O)O)N)S', 'CCC(C)C(C(=O)O)N', 'C1=C(NC=N1)C(C(=O)O)N', 'CSCCC(C(=O)O)N', 'C1=CC=C(C=C1)CC(C(=O)O)N', 'C(C(C(=O)O)N)C(=O)N', 'C1=C(NC=N1)C(C(=O)O)N', 'C1CC(NC1)C(=O)O', 'C(CC(=O)N)(C(=O)O)N', 'C(C(C(=O)O)N)C(=O)N', 'C(C(C(=O)O)N)C(=O)O', 'C(C(C(=O)O)N)S']	'cysteine isoleucine histidine methionine phenylalanine asparagine histidine proline glutamine asparagine aspartate cysteine'
HPQ12	CPHLSKAHPQVC	N[C@@]([H])(CS)C(=O)N1[C@@]([H])(CCC1)C(=O)N[C@@]([H])(CC1=CN=C-N1)C(=O)N[C@@]([H])(CC(C)C)C(=O)N[C@@]([H])(CO)C(=O)N[C@@]([H])(CCCCN)C(=O)N[C@@]([H])(C)C(=O)N[C@@]([H])(CC1=CN=C-N1)C(=O)N1[C@@]([H])(CCC1)C(=O)N[C@@]([H])(CCC(=O)N)C(=O)N[C@@]([H])(C(C)C)C(=O)N[C@@]([H])(CS)C(=O)O	['C(C(C(=O)O)N)S', 'C1CC(NC1)C(=O)O', 'C1=C(NC=N1)C(C(=O)O)N', 'CC(C)CC(C(=O)O)N', 'C(C(C(=O)O)N)O', 'C(CCN)CC(C(=O)O)N', 'CC(C(=O)O)N', 'C1=C(NC=N1)C(C(=O)O)N', 'C1CC(NC1)C(=O)O', 'C(CC(=O)N)(C(=O)O)N', 'CC(C)C(C(=O)O)N', 'C(C(C(=O)O)N)S']	'cysteine proline histidine leucine serine lysine alanine histidine proline glutamine valine cysteine'

Supplementary Table 2: Peptide sequences for CCA cyclized peptides, Str1-9

Peptide name	Peptide sequence	Peptide sequence SMILES	Peptide sequence SMILES positional	Peptide sequence text
Str1	CPINIFHPPPDC	N[C@@]([H])(CS)C(=O)N1[C@@]([H])(CCC1)C(=O)N[C@@]([H])([C@]([H])(CC)C)C(=O)N[C@@]([H])(CC(=O)N)C(=O)N[C@@]([H])([C@]([H])(CC)C)C(=O)N[C@@]([H])(Cc1ccccc1)C(=O)N[C@@]([H])(CC1=CN=C-N1)C(=O)N1[C@@]([H])(CCC1)C(=O)N1[C@@]([H])(CCC1)C(=O)N1[C@@]([H])(CCC1)C(=O)N[C@@]([H])(CC(=O)O)C(=O)N[C@@]([H])(CS)C(=O)O	['C(C(C(=O)O)N)S', 'C1CC(NC1)C(=O)O', 'CCC(C)C(C(=O)O)N', 'C(C(C(=O)O)N)C(=O)N', 'CCC(C)C(C(=O)O)N', 'C1=CC=C(C=C1)CC(C(=O)O)N', 'C1=C(NC=N1)C(C(=O)O)N', 'C1CC(NC1)C(=O)O', 'C1CC(NC1)C(=O)O', 'C1CC(NC1)C(=O)O', 'C(C(C(=O)O)N)C(=O)O', 'C(C(C(=O)O)N)S']	'cysteine proline isoleucine asparagine isoleucine phenylalanine histidine proline proline proline aspartate cysteine'
Str2	CPFNIKNPDDGC	N[C@@]([H])(CS)C(=O)N1[C@@]([H])(CCC1)C(=O)N[C@@]([H])(Cc1ccccc1)C(=O)N[C@@]([H])(CC(=O)N)C(=O)N[C@@]([H])([C@]([H])(CC)C)C(=O)N[C@@]([H])(CCCCN)C(=O)N[C@@]([H])(CC(=O)N)C(=O)N1[C@@]([H])(CCC1)C(=O)N[C@@]([H])(CC(=O)O)C(=O)N[C@@]([H])(CC(=O)O)C(=O)NCC(=O)N[C@@]([H])(CS)C(=O)O	['C(C(C(=O)O)N)S', 'C1CC(NC1)C(=O)O', 'C1=CC=C(C=C1)CC(C(=O)O)N', 'C(C(C(=O)O)N)C(=O)N', 'CCC(C)C(C(=O)O)N', 'C(CCN)CC(C(=O)O)N', 'C(C(C(=O)O)N)C(=O)N', 'C1CC(NC1)C(=O)O', 'C(C(C(=O)O)N)C(=O)O', 'C(C(C(=O)O)N)C(=O)O', 'C(C(=O)O)N', 'C(C(C(=O)O)N)S']	'cysteine proline phenylalanine asparagine isoleucine lysine asparagine proline aspartate aspartate glycine cysteine'
Str3	CPYNIAGPPLYC	N[C@@]([H])(CS)C(=O)N1[C@@]([H])(CCC1)C(=O)N[C@@]([H])(Cc1ccc(O)cc1)C(=O)N[C@@]([H])(CC(=O)N)C(=O)N[C@@]([H])([C@]([H])(CC)C)C(=O)N[C@@]([H])(C)C(=O)NCC(=O)N1[C@@]([H])(CCC1)C(=O)N1[C@@]([H])(CCC1)C(=O)N[C@@]([H])(CC(C)C)C(=O)N[C@@]([H])(Cc1ccc(O)cc1)C(=O)N[C@@]([H])(CS)C(=O)O	['C(C(C(=O)O)N)S', 'C1CC(NC1)C(=O)O', 'C1=CC(=CC=C1CC(C(=O)O)N)O', 'C(C(C(=O)O)N)C(=O)N', 'CCC(C)C(C(=O)O)N', 'CC(C(=O)O)N', 'C(C(=O)O)N', 'C1CC(NC1)C(=O)O', 'C1CC(NC1)C(=O)O', 'CC(C)CC(C(=O)O)N', 'C1=CC(=CC=C1CC(C(=O)O)N)O', 'C(C(C(=O)O)N)S']	'cysteine proline tyrosine asparagine isoleucine alanine glycine proline proline leucine tyrosine cysteine'
Str4	CPFNILVPFIDC	N[C@@]([H])(CS)C(=O)N1[C@@]([H])(CCC1)C(=O)N[C@@]([H])(Cc1ccccc1)C(=O)N[C@@]([H])(CC(=O)N)C(=O)N[C@@]([H])([C@]([H])(CC)C)C(=O)N[C@@]([H])(CC(C)C)C(=O)N[C@@]([H])(C(C)C)C(=O)N1[C@@]([H])(CCC1)C(=O)N[C@@]([H])(Cc1ccccc1)C(=O)N[C@@]([H])([C@]([H])(CC)C)C(=O)N[C@@]([H])(CC(=O)O)C(=O)N[C@@]([H])(CS)C(=O)O	['C(C(C(=O)O)N)S', 'C1CC(NC1)C(=O)O', 'C1=CC=C(C=C1)CC(C(=O)O)N', 'C(C(C(=O)O)N)C(=O)N', 'CCC(C)C(C(=O)O)N', 'CC(C)CC(C(=O)O)N', 'CC(C)C(C(=O)O)N', 'C1CC(NC1)C(=O)O', 'C1=CC=C(C=C1)CC(C(=O)O)N', 'CCC(C)C(C(=O)O)N', 'C(C(C(=O)O)N)C(=O)O', 'C(C(C(=O)O)N)S']	'cysteine proline phenylalanine asparagine isoleucine leucine valine proline phenylalanine isoleucine aspartate cysteine'
Str5	CPYNIINPTFDC	N[C@@]([H])(CS)C(=O)N1[C@@]([H])(CCC1)C(=O)N[C@@]([H])(Cc1ccc(O)cc1)C(=O)N[C@@]([H])(CC(=O)N)C(=O)N[C@@]([H])([C@]([H])(CC)C)C(=O)N[C@@]([H])([C@]([H])(CC)C)C(=O)N[C@@]([H])(CC(=O)N)C(=O)N1[C@@]([H])(CCC1)C(=O)N[C@@]([H])([C@]([H])(O)C)C(=O)N[C@@]([H])(Cc1ccccc1)C(=O)N[C@@]([H])(CC(=O)O)C(=O)N[C@@]([H])(CS)C(=O)O	['C(C(C(=O)O)N)S', 'C1CC(NC1)C(=O)O', 'C1=CC(=CC=C1CC(C(=O)O)N)O', 'C(C(C(=O)O)N)C(=O)N', 'CCC(C)C(C(=O)O)N', 'CCC(C)C(C(=O)O)N', 'C(C(C(=O)O)N)C(=O)N', 'C1CC(NC1)C(=O)O', 'C(C(C(=O)O)N)(O)C', 'C1=CC=C(C=C1)CC(C(=O)O)N', 'C(C(C(=O)O)N)C(=O)O', 'C(C(C(=O)O)N)S']	'cysteine proline tyrosine asparagine isoleucine isoleucine asparagine proline threonine phenylalanine aspartate cysteine'
Str6	CPFNIASPFYGC	N[C@@]([H])(CS)C(=O)N1[C@@]([H])(CCC1)C(=O)N[C@@]([H])(Cc1ccccc1)C(=O)N[C@@]([H])(CC(=O)N)C(=O)N[C@@]([H])([C@]([H])(CC)C)C(=O)N[C@@]([H])(C)C(=O)N[C@@]([H])(CO)C(=O)N1[C@@]([H])(CCC1)C(=O)N[C@@]([H])(Cc1ccccc1)C(=O)N[C@@]([H])(Cc1ccc(O)cc1)C(=O)NCC(=O)N[C@@]([H])(CS)C(=O)O	['C(C(C(=O)O)N)S', 'C1CC(NC1)C(=O)O', 'C1=CC=C(C=C1)CC(C(=O)O)N', 'C(C(C(=O)O)N)C(=O)N', 'CCC(C)C(C(=O)O)N', 'CC(C(=O)O)N', 'C(C(C(=O)O)N)O', 'C1CC(NC1)C(=O)O', 'C1=CC=C(C=C1)CC(C(=O)O)N', 'C1=CC(=CC=C1CC(C(=O)O)N)O', 'C(C(=O)O)N', 'C(C(C(=O)O)N)S']	'cysteine proline phenylalanine asparagine isoleucine alanine serine proline phenylalanine tyrosine glycine cysteine'
Str7	CPINVQYSTGTC	N[C@@]([H])(CS)C(=O)N1[C@@]([H])(CCC1)C(=O)N[C@@]([H])([C@]([H])(CC)C)C(=O)N[C@@]([H])(CC(=O)N)C(=O)N[C@@]([H])(C(C)C)C(=O)N[C@@]([H])(CCC(=O)N)C(=O)N[C@@]([H])(Cc1ccc(O)cc1)C(=O)N[C@@]([H])(CO)C(=O)N[C@@]([H])([C@]([H])(O)C)C(=O)NCC(=O)N[C@@]([H])([C@]([H])(O)C)C(=O)N[C@@]([H])(CS)C(=O)O	['C(C(C(=O)O)N)S', 'C1CC(NC1)C(=O)O', 'CCC(C)C(C(=O)O)N', 'C(C(C(=O)O)N)C(=O)N', 'CC(C)C(C(=O)O)N', 'C(CC(=O)N)(C(=O)O)N', 'C1=CC(=CC=C1CC(C(=O)O)N)O', 'C(C(C(=O)O)N)O', 'C(C(C(=O)O)N)(O)C', 'C(C(=O)O)N', 'C(C(C(=O)O)N)(O)C', 'C(C(C(=O)O)N)S']	'cysteine proline isoleucine asparagine valine glutamine tyrosine serine threonine glycine threonine cysteine'

Str8	CPYNVERPD SHC	N[C@@]([H])(CS)C(=O)N1[C@@]([H])(CCC1)C(=O)N[C@@]([H])(Cc1ccc(O)cc1)C(=O)N[C@@]([H])(CC(=O)N)C(=O)N[C@@]([H])(C(C)C)C(=O)N[C@@]([H])(CCC(=O)O)C(=O)N[C@@]([H])(CCCNC(=N)N)C(=O)N1[C@@]([H])(CCC1)C(=O)N[C@@]([H])(CC(=O)O)C(=O)N[C@@]([H])(CO)C(=O)N[C@@]([H])(CC1=CN=C-N1)C(=O)N[C@@]([H])(CS)C(=O)O	['C(C(C(=O)O)N)S', 'C1CC(NC1)C(=O)O', 'C1=CC(=CC=C1CC(C(=O)O)N)O', 'C(C(C(=O)O)N)C(=O)N', 'CC(C)C(C(=O)O)N', 'C(CC(=O)O)(C(=O)O)N', 'C(C(C(=O)O)N)CN=C(N)N', 'C1CC(NC1)C(=O)O', 'C(C(C(=O)O)N)C(=O)O', 'C(C(C(=O)O)N)O', 'C1=C(NC=N1)C(C(=O)O)N', 'C(C(C(=O)O)N)S']	'cysteine proline tyrosine asparagine valine glutamate arginine proline aspartate serine histidine cysteine'
Str9	CPFNFFIWND EC	N[C@@]([H])(CS)C(=O)N1[C@@]([H])(CCC1)C(=O)N[C@@]([H])(Cc1ccccc1)C(=O)N[C@@]([H])(CC(=O)N)C(=O)N[C@@]([H])(Cc1ccccc1)C(=O)N[C@@]([H])(Cc1ccccc1)C(=O)N[C@@]([H])([C@]([H])(CC)C)C(=O)N[C@@]([H])(CC(=CN2)C1=C2C=CC=C1)C(=O)N[C@@]([H])(CC(=O)N)C(=O)N[C@@]([H])(CC(=O)O)C(=O)N[C@@]([H])(CCC(=O)O)C(=O)N[C@@]([H])(CS)C(=O)O	['C(C(C(=O)O)N)S', 'C1CC(NC1)C(=O)O', 'C1=CC=C(C=C1)CC(C(=O)O)N', 'C(C(C(=O)O)N)C(=O)N', 'C1=CC=C(C=C1)CC(C(=O)O)N', 'C1=CC=C(C=C1)CC(C(=O)O)N', 'CCC(C)C(C(=O)O)N', 'C1=CC=C2C(=C1)C(=CN2)CC(C(=O)O)N', 'C(C(C(=O)O)N)C(=O)N', 'C(C(C(=O)O)N)C(=O)O', 'C(CC(=O)O)(C(=O)O)N', 'C(C(C(=O)O)N)S']	'cysteine proline phenylalanine asparagine phenylalanine phenylalanine isoleucine tryptophan asparagine aspartate glutamate cysteine'

4. Supplementary References

(1) Istrate, A.; Geeson, M. B.; Navo, C. D.; Sousa, B. B.; Marques, M. C.; Taylor, R. J.; Journeaux, T.; Oehler, S. R.; Mortensen, M. R.; Deery, M. J. Platform for orthogonal N-cysteine-specific protein modification enabled by cyclopropenone reagents. *J. Am. Chem. Soc.* **2022**, *144* (23), 10396-10406.

(2) Li, K.; Wang, W.; Gao, J. Fast and Stable N-Terminal Cysteine Modification through Thiazolidino Boronate Mediated Acyl Transfer. *Angew. Chem., Int. Ed..* **2020**, *59* (34), 14246-14250.
